# *Lactobacillus rhamnosus* GG as an Effective Probiotic for Murine Giardiasis

**DOI:** 10.1155/2011/795219

**Published:** 2011-06-02

**Authors:** Nisha Goyal, Ram Prakash Tiwari, Geeta Shukla

**Affiliations:** Department of Microbiology, Panjab University, Chandigarh 160014, India

## Abstract

The gut microflora is an important constituent in the intestinal mucosal barrier and has been introduced as the concept of probiotic therapy that beneficially affects the host by improving its intestinal microbial balance. Therefore, the main objective of the study was to explore the protective potential of various lactobacilli strains for murine giardiasis. By experimentation, it was found that the probiotic supplementation of either *Lactobacillus casei*, *L. acidophilus*, *L. plantarum*, or *L. rhamnosus* GG, 7 days prior to inoculation with *G. lamblia* trophozoites, reduced the rate of cyst excretion compared with *Giardia*-infected mice. Interestingly, *L.* GG was found to be the most effective probiotic in reducing the duration of giardia cycle and acts as an effective prophylactic probiotic for murine giardiasis but needs to be clinically correlated due to entirely different human microflora.

## 1. Introduction

The zoonotic intestinal disease giardiasis is caused by the enteric protozoan parasite *Giardia lamblia*, one of the most common causes of intestinal infections worldwide. Infection with *Giardia* is acquired by the ingestion of viable cysts due to inadequate sanitation or poor treatment of drinking water. Giardiasis is a disease of main concern as it affects children, adults, hypogammaglobulineamic, malnourished, and immunocompromised individuals leading to either acute or chronic diarrhea, nausea, malabsorption, weight loss, steatorrhoea, and growth retardation particularly in young children [1–3]. The first line of treatment for giardiasis is antibiotic like nitroimidazoles and nitrofurans. However, antibiotic therapy is associated with many unpleasant side effects (e.g., metallic taste), poor patient compliance, and enhanced occurrence of resistance leading to subsequent treatment failure. Thus, this has encouraged research on alternative biotherapeutic strategies such as plant extracts (phytomedicine), products derived from bees, and probiotics that are safe, inexpensive, and effective in improving the cause of intestinal parasitosis [4–6]. 

Normally, the generation of immunophysiologic regulation in the gut depends on the establishment of indigenous microflora and has led to the introduction of novel therapeutic interventions based on the consumption of cultures of beneficial live microorganisms, the probiotics [7]. Probiotics are live microorganisms that beneficially affect the gastrointestinal balance if ingested in sufficient numbers, and provide health benefits that go beyond normal nutritional effects [8]. Probiotics, also known as microbial interference therapy (MIT), offer an attractive supportive therapy for gastrointestinal infections by acting as surrogative normal flora via various mechanisms such as production of antimicrobial substances, modification of toxins, interference with attachment, stimulation of immune system or a combination of mechanisms [9, 10]. In our earlier studies, we have observed that the probiotics *L. casei* and *L. acidophilus* both modulate the murine giardiasis by reducing the severity and duration of the disease [11–13]. However, it has also been observed that different strains of lactic acid bacteria (LAB) have different properties and may beneficially influence the composition and metabolic activity of the endogenous microbiota [7, 14] or inhibit the growth of a wide range of enteropathogens in intestinal diseases [15], as not all the strains are able to adequately survive the acidic pH or to adhere and colonize the gut. Moreover, till date no effective probiotic has been reported for giardiasis, thus it is pertinent to delineate an effective probiotic for giardiasis.

## 2. Methods

### 2.1. Parasite and Culture Conditions

*Giardia lamblia* trophozoites (Portland strain I) were grown axenically in TYI-S-33 medium supplemented with antibiotic solution, and pH was adjusted to 6.9 before sterilization with 0.22 *μ*m seitz filter. For experimental inoculation, actively growing trophozoites (48–72 h old culture) were sedimented after chilling the tubes in ice for 15 min and finally suspended in phosphate buffer saline (PBS-7.2) to contain 1 × 10^6^ trophozoites/0.1 mL [12].

### 2.2. Bacterial Strains, Preparation, and Inoculation

Various lactobacilli strains (*Lactobacillus* GG, *L. acidophilus*, *L. plantarum*, and *L. casei*) were procured from Microbial Type Culture Collection (MTCC), Institute of Microbial Technology (IMTECH), Chandigarh, India. These lactobacilli* strains *were grown in MRS medium for 18 hours. Thereafter, the cultures were centrifuged, washed, and suspended in PBS-7.2 to contain 1 × 10^9^ lactobacilli/0.1 mL and were fed via orogastric gavage [12].

### 2.3. Animals

BALB/c mice aged 5-6 weeks old (18–20 gm) were obtained from Central Animal House, Panjab University, Chandigarh, India. These were housed under standard conditions of light and dark cycle and were fed with laboratory diet and water *ad libitum*. Water and feed before supplementation to animals were monitored for any bacterial or parasitic contamination by Gram's staining and Lugol's iodine staining techniques [16]. Animals were also screened for *Giardia* infection via simple microscopic stool examination for three consecutive days. Only *Giardia*-free mice were employed. Care and use of animals were in accordance with the guidelines of the institutional ethical committee.

### 2.4. Experimental Design and Followup of the Animals

 Animals were divided mainly into five groups. Each group comprised of 6 animals. Group I (*Giardia* infected): these mice were challenged orally with a single dose of 1 × 10^6^ *Giardia* trophozoites via orogastric gavage. Group II (*L. casei-Giardia*); Group III (*L. acidophilus- Giardia*); Group IV (*L. plantarum-Giardia*); Group V (*L. rhamnosus* GG- *Giardia*). Animals belonging to Groups II, III, IV, and V were fed orally with a single dose of respective lactobacilli strains (1 × 10^9^ lactobacilli/0.1 mL) for 7 days. On the 8th day, a single challenge dose of *Giardia* trophozoites (1 × 10^6^ trophozoites) was given orally along with a single dose of probiotic treatment. The probiotic treatment was further continued for 25 days [11]. After respective treatments in all the groups, *Giardia *cyst count was monitored. Lactobacilli and trophozoite counts were monitored only with the most effective probiotics.

### 2.5. Giardia Cysts in Faeces

Briefly, one gram of freshly passed faecal samples was dissolved in 10 mL of normal saline and homogenized using pestle and mortar mixer. Slide was prepared, and cysts stained with iodine were counted on every third day using a hemocytometer and were expressed as cysts mL^−1^ [11].

### 2.6. Lactobacilli in Faeces with *L.* GG

To confirm if the lactobacilli species were able to survive the stress and colonize within the gastrointestinal tract, freshly voided faecal samples of mice belonging to Groups I and V were homogenized in normal saline and serially diluted. The diluted homogenates (0.1 mL) were spread plated on MRS agar and incubated at 37°C for 24–48 hrs, and CFU were counted [11].

### 2.7. Giardia Trophozoites in the Small Intestine (Jejunum) with *L.* GG as the Probiotic

 Mice were sacrificed, and the proximal 10 cm section, mainly the jejunum, was removed and placed in 5 mL of the ice-chilled isotonic saline solution. The small intestine sections were minced and kept for 15–20 minutes in ice-chilled saline, and trophozoites were counted using a haemocytometer. Mice with no detectable trophozoites were considered to have cleared the parasite infection [12].

### 2.8. Statistical Analysis

Results were expressed as mean ± SD. The inter-group variation was assessed by student's *t*-test and one way analysis of variance (ANOVA) with equal number of observations followed by Tukey's multiple comparison test. Statistical significance of the result was calculated at *P* < .05.

## 3. Results

### 3.1. Giardia Cycle

**Figure 1:**
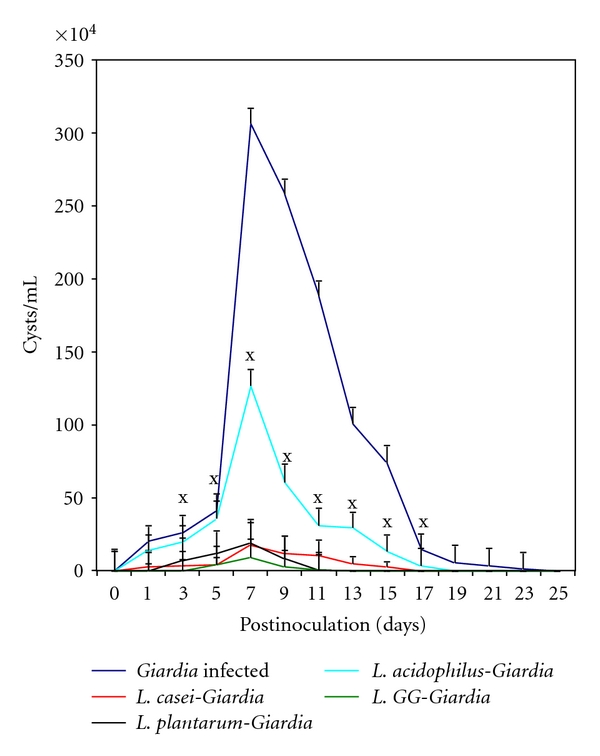
*Giardia* cysts in faeces of mice belonging to different groups. Values are mean ± SD, *P < .05 versus* Giardia*.

*Giardia*-infected mice (Group I) voided cysts gradually from day 1 onwards and was significantly (*P* < .05) highest (306.4 × 10^4^ ± 10.63) on day 7 postinoculation (PI). Thereafter, the cyst count started decreasing, and mice became *Giardia*-free by day 25 PI (Figure 1). However, oral feeding either with *L. acidophilus*, *L. plantarum*, *L. casei*, *L. acidophilus*, or *L. *GG significantly (*P* < .05) reduced the cyst excretion in mice belonging to all the groups (II, III, IV, and V) from the beginning and became *Giardia-*free by days 17 PI (Groups II and III) and 13 PI (Groups IV and V), respectively. Interestingly, mice fed orally with *L. GG *(Group V) showed significantly (*P* < .05) least cyst count from the beginning of the infection (i.e., 5 days PI onwards) and became *Giardia*-free by day 13 PI (Figure 1). However, none of the mice from any of these groups showed any clinical symptoms like diarrhea, weight loss, and death.

### 3.2. Lactobacilli in Faeces with *L.* GG

**Figure 2:**
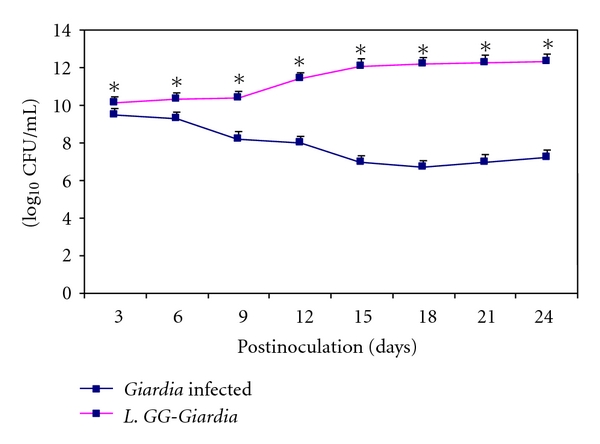
Lactobacilli count (log_10_ CFU/mL) in faeces. Values are mean ± SD, * *P* < .05 versus* Giardia*.

The faecal lactobacilli counts increased significantly (*P* < .05) in* L. GG-Giardia *mice (Group V) from the beginning and were significantly higher (*P* < .05) at each point of observation compared with *Giardia-*infected mice (Group I) that had least lactobacilli count (Figure 2).

### 3.3. Giardia Trophozoites in the Intestinal Fluid with *L.* GG

**Figure 3:**
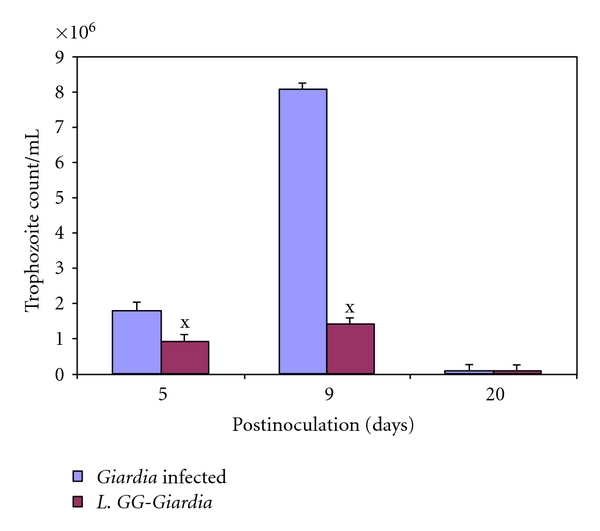
*Giardia* trophozoites count in the small intestine. Values are mean ± SD, * *P* < .05 versus* Giardia*.

 It was found that oral administration of *Giardia* trophozoites resulted in the establishment of infection as assessed by the number of trophozoites in the jejunum and cyst counts in faeces. Interestingly, trophozoite counts were significantly (*P* < .05) reduced in the gut of *L. GG*-treated mice (Group V) compared with Giardia*-*infected mice (Group I, Figure 3).

## 4. Discussion

Giardiasis is a diarrheal disease mainly of young children and immunosuppressed or malnourished individuals. It is generally accepted that the enteric bacterial environment represents a physiological factor that can interfere with the process of a *G. lamblia* infection. Therefore, the present study was designed to assess the effect of various probiotic supplementations in modulating the *Giardia* cycle in BALB/c mice. It was found that orally administered *Giardia* trophozoites in mice could transiently colonize the gut, and infection was self-limiting. It is very well evident that the supplementation of various probiotics has the potential to modulate murine giardiasis to variable extent. Amongst all the lactobacilli, *L. GG* was found to be the most effective probiotic in modulating the giardiasis, both in terms of duration of *Giardia* cycle and rate of cyst excretion. The present observation of reduced cyst count may be either due to better survival of *L. GG* in stomach or effective adherence and colonization in the gut compared with other lactobacilli strains [17]. Moreover, *L. GG* has also been found to be an effective treatment therapy for various bacterial and viral diarrheal diseases and is in accordance with the earlier studies [8, 11–13]. 

The ability of the *L. GG* to adhere to gastrointestinal tract and persistence was further evaluated by monitoring the lactobacilli count in faeces. It was observed that mice fed with *L. GG* had more lactobacilli in faeces compared with Giardia-infected mice alone. Interestingly, it was also found that *L. GG* feeding reduced the active number of Giardia trophozoites in the gut leading to early resolution of Giardia infection by day 13 (PI). This may again be suggestive of effective colonizing ability of *L. GG* and interaction with enterocytes, thus enriching the endogenous microbiota and is in accordance with earlier studies [11, 18–20]. The reduced duration of infection may be partly either due to effective colonization of lactobacilli, production of secretary substance or competition for nutrients, and so forth. However, the modification of other key intestinal components remains to be screened. 

Taken together, it can be concluded that various lactobacilli species have variable effects in intestinal diseases. Among the four lactobacilli species, *L. GG *was found to be the most effective probiotics for murine giardiasis. Thus it can be said that *L. GG *is the ideal, safe, and stronger barrier against intestinal pathogens than other lactobacilli strains, leading to improved core health, that is, healthier digestion and improved immune system, and it may also serve as an alternative mode for the prevention of giardiasis.

##  Conflict of Interests

The authors report no conflict of interests. They alone are responsible for the content and writing of the paper.
